# Basal Cell Carcinoma of the Penis: A Case Report and Review of the Literature

**DOI:** 10.1155/2014/173076

**Published:** 2014-09-14

**Authors:** R. J. Roewe, Matthew A. Uhlman, Nathan A. Bockholt, Amit Gupta

**Affiliations:** Department of Urology, University of Iowa, 200 Hawkins Drive, 3 Roy Carver Pavilion, Iowa City, IA 52242-1089, USA

## Abstract

Basal cell carcinoma of the penis is an extremely rare entity, accounting for less than 0.03% of all basal cell carcinomas. Fortunately, wide local excision of such lesions is generally curative. Fewer than 25 cases have been reported in the literature describing penile basal cell carcinoma. Here we report a case of penile basal cell carcinoma cured with wide local excision.

## 1. Introduction

Basal cell carcinoma (BCC) is the most common malignancy of the skin, accounting for approximately 80% of all skin cancers [1]. Ultraviolet (UV) radiation exposure, in combination with a multitude of other established risk factors including age, Caucasian race, male gender, and immunosuppression, has historically type-cast BCC as a cancer involving sun-exposed skin areas [1–3]. However, numerous reports of BCC in nonsun-exposed sites, such as the penile, scrotal, and perianal region, have been documented and have inspired a search for other etiologic factors [4].

BCC of the penis accounts for only 0.01–0.03% of all BCC's in men [5, 6]. As of 2006, there were only twenty-three reported cases of penile BCC. These cases may tend to present later and require more invasive surgical intervention compared to BCC at other skin regions, leading to excessive physiological and psychological morbidity related to surgical treatment [5, 6]. Here, we report a case of penile BCC at the penile base in a middle-aged Caucasian male that was successfully treated with wide local excision.

## 2. Case Presentation

A 56-year-old Caucasian male presented with a one-centimeter ulcerating lesion on the left base of the penis present for approximately one year. Of note, he also had a perianal papillary lesion that had been present for several years, but no appreciable inguinal lymphadenopathy. Sexually transmitted disease screening was negative. No other tests or imaging were undertaken at that time. His past medical history was significant for tobacco use and a distant history of gonorrhea infection several decades before. He denied any history of skin cancer, other familial cancers, or abnormal skin exposures.

**Figure 1:**
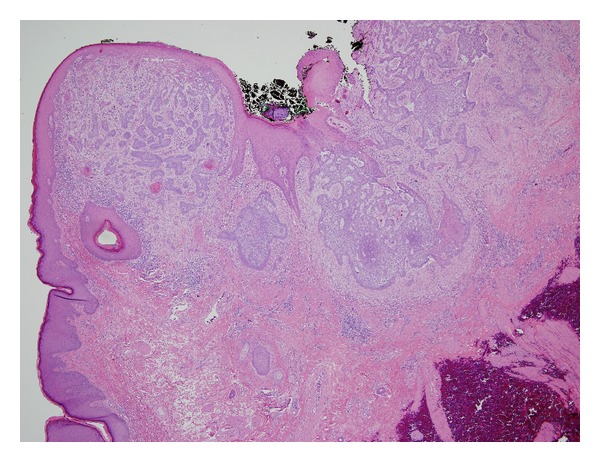
Hematoxylin and eosin stain of lesion.

**Figure 2:**
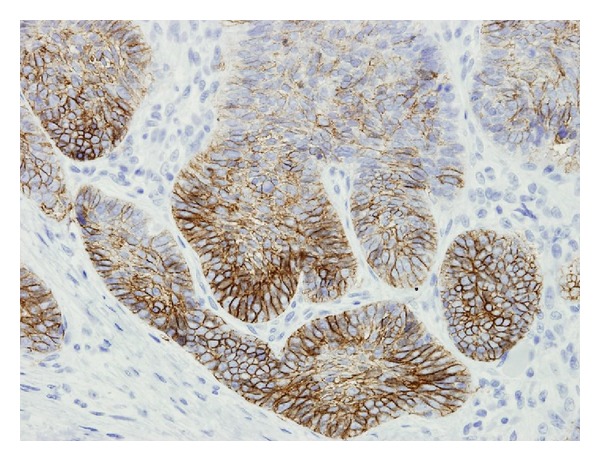
Basal cell tumor showing immunoreactivity with keratin 34bE12 antibody.

The penile lesion was surgically excised in clinic under local anesthesia with approximately 0.5 cm margins. Grossly the specimen was described as an irregular red-brown lesion, measuring 2.2 × 1.0 cm. Surgical pathology revealed basal cell carcinoma, with infiltrative features (Figure 1). The tumor cells were positive for Ber-Ep4 on immunohistochemical staining (Figure 2). The perianal lesion was also removed and found to be a skin tag. The patient had an uneventful course following the procedure and had a minimal pain and is currently doing well. His penis has completely healed and is fully functional, with no signs of recurrence.

## 3. Discussion

Penile cancer is an extremely rare malignancy, accounting for only 0.4–0.6% of all malignancies in the United States and Western Europe [3]. The majority of these cancers, approximately 95%, are squamous cell carcinoma (SCC) and are associated with HPV infection, poor hygiene, lack of circumcision, phimosis, and lichen sclerosis [1, 7]. BCC, along with melanoma, extra mammary Paget's disease, and soft tissue sarcomas, accounts for the other five percent of penile malignancies and has not been well characterized.

BCC, in general, is a relatively slow growing entity that emerges from the epidermis and most commonly occurs in the fifth to seventh decades of life [4, 5]. Classically, it is described as a raised pearly lesion with rolled borders and telangiectasias with or without ulceration. It has a low incidence of metastasis, between 0.003 and 0.1% as it characteristically spreads by slow, local invasion [5]. The most common treatment is surgical by wide local excision or Mohs micrographic surgery. However, topical therapies with imiquimod or fluorouracil are other potential first-line treatment options [1].

The pathogenesis of BCC in sun-exposed areas is associated with intense and intermittent UV radiation exposure, particularly in childhood and adolescence [1, 2]. Male gender, age, Caucasian descent, immunosuppression, and previous radiation are other identifiable markers that have been shown to be associated with a higher risk BCC [1–3]. The occurrence of BCC on nonsun-exposed areas implicates other possible etiologies in the development of BCC. Gibson and Ahmed retrospectively examined 51 cases of perianal and genital basal cell carcinoma in men and women and found that only 36% had a history of nonmelanoma skin cancer in other areas, 8.5% had prior pelvic radiation and 4.25% had received immunosuppressive drugs in their recent past [4]. A multitude of other reports have failed to identify new risk factors associated with these cases of penile BCC. Of note, several reports have observed that these lesions tend to present later than expected, as the average size at presentation is approximately 2 centimeters with ulceration often present [4–6]. Accordingly, there is growing concern of delayed diagnoses in these patients, often after treatment failure for other presumed dermatological diagnosis (i.e., inflammatory, infectious, allergic, etc.). This delay leads to more extensive surgical morbidity and possibly to an increased risk of recurrence or metastatic spread [4–6].

Our patient holds several of the classic identifiable traits placing him at higher risk of BCC—male gender, middle-age, and Caucasian descent—but no history of other skin malignancies, immunosuppression, or previous radiation. He does have a significant history of tobacco use, as well as a distant history of a gonorrhea infection, but no other significant past medical, surgical, or family history. To our knowledge, no association between smoking or previous STD's and BCC has been established.

In terms of treatment, surgical management with wide local excision at initial presentation was curative without postoperative complications. Early and aggressive surgical management allowed for minimal surgical morbidity and a successful outcome—the ultimate goal when managing lesions involving such psychosocial sensitive areas.

## 4. Conclusion

Basal cell carcinoma of the penis is an extremely rare entity with an excellent prognosis. Unfortunately, patients may tend to present later with lesions involving this region, resulting in more extensive surgical management that leads to significant morbidity, both physical and psychological. Early diagnosis and management are instrumental in avoiding such morbidity as well as minimizing the risk of recurrence or metastatic spread for the treatment of penile BCC.

## References

[1] Firnhaber JM (2012). Diagnosis and treatment of basal cell and squamous cell carcinoma. *The American Family Physician*.

[2] Kudchadkar R, Lewis K, Gonzalez R (2012). Advances in the treatment of basal cell carcinoma: hedgehog inhibitors. *Seminars in Oncology*.

[3] Brady KL, Mercurio MG, Brown MD (2013). Malignant tumors of the penis. *Dermatologic Surgery*.

[4] Gibson GE, Ahmed I (2001). Perianal and genital basal cell carcinoma: a clinicopathologic review of 51 cases. *Journal of the American Academy of Dermatology*.

[5] Lidder S, Lang KJ, Nakhdjevani A (2011). Basal cell carcinoma of the penis. *Singapore Medical Journal*.

[6] Nguyen H, Saadat P, Bennett RG (2006). Penile basal cell carcinoma: two cases treated with Mohs micrographic surgery and remarks on pathogenesis. *Dermatologic Surgery*.

[7] Barnes KT, Smith BJ, Lynch CF, Gupta A (2013). Obesity and invasive penile cancer. *European Urology*.

